# Use of Community Treatment Orders in Inpatient Rehabilitation Services in Leicester, Leicestershire and Rutland: Findings From the Leicestershire Inpatient Rehabilitation Outcomes (LIRO) Research

**DOI:** 10.1192/bjo.2025.10220

**Published:** 2025-06-20

**Authors:** Edvin Sajquim, Kelly Fenton, Katherine Kidd, Sandeep Singh

**Affiliations:** Leicestershire Partnership NHS Trust, Leicester, United Kingdom

## Abstract

**Aims:** 1. To evaluate and understand the sociodemographic characteristics of the patients admitted to rehabilitation wards who were subjected to CTO. 2. To evaluate the rehabilitation of inpatients discharged with or without CTO in place and compare their readmission rate.

**Methods:** The LIRO is the biggest database of inpatients in rehabilitation services in the NHS to our knowledge. The data is collected from admissions to inpatient mental health rehabilitation services between 2014 and 2020. The inpatient services involved two rehabilitation hospitals in Leicestershire, with a total of 68 beds.

**Results:** 361 admissions were identified. 72 (19.94%) patients were discharged on a CTO and 289 (80.05%) patients were discharged without CTO. 21% of the total of male patients (n=46) were discharged on a CTO vs 17.01% (n=25) of the female patients, one transgender patient was discharged on a CTO (p=0.311). Demographically, 34 (14.47%) Caucasian patients were discharged under CTO while 37 (30.33%) were non-Caucasian (p<0.001). 49 (68.05%) of the patients on CTO were discharged on depot antipsychotic as treatment, compared with 72 (24%) of patients without a CTO (p<0.0001).

Upon one year follow up, 32 (44.44%) patients on CTO were readmitted to mental health services within 12 months, while 126 patients discharged without CTO were readmitted within 12 months of discharge (43.60%) (p=0.97).

**Conclusion:** Patients subjected to the CTO are more likely to be discharged on depot medications from rehabilitation services. Significant number of non-Caucasian patients in rehabilitation services tend to be discharged on CTO. This is similar to national findings. There is no difference identified in readmission rate after discharge from rehabilitation ward within one year between the patients on CTO or not. This adds to the growing evidence that CTO is not stopping revolving door presentation.

